# Storage Behavior and Response to Low-Cost Postharvest Technologies of the Underutilized Purple Yampee (*Dioscorea trifida* L.f.)

**DOI:** 10.3390/foods14142436

**Published:** 2025-07-10

**Authors:** Sandra Viviana Medina-López, Jorge Andrés Jola Hernández, Maria Soledad Hernández-Gómez, Juan Pablo Fernández-Trujillo

**Affiliations:** 1 Instituto de Ciencia y Tecnología de Alimentos (ICTA), Universidad Nacional de Colombia, Bogotá 111321, Colombia; svmedinal@unal.edu.co (S.V.M.-L.); mshernandez@unal.edu.co (M.S.H.-G.); 2 Departamento de Ingeniería Agronómica, Universidad Politécnica de Cartagena, 30203 Cartagena, Spain; 3 Facultad de Ciencias Agrarias, Universidad Nacional de Colombia, Bogotá 111321, Colombia; jjola@unal.edu.co; 4 Instituto Amazónico de Investigaciones Científicas SINCHI, Bogotá 110311, Colombia

**Keywords:** tuber, shelf-life, biodiversity, antioxidants, edible coatings, calcium chloride, hot water dips

## Abstract

Postharvest losses and limited physiological knowledge restrict the conservation and year-round availability of underutilized crops such as *Dioscorea trifida*. This study characterized the postharvest behavior of Colombian purple *D. trifida* tubers and evaluated low-cost, GRAS-status technologies to improve storage performance in smallholder production systems. Tubers were stored for 34 days at ambient conditions (20 °C, 90% RH) and compared with treatments including cold storage, calcium pretreatments combined with *Aloe vera*-based coatings, and short-duration hot water immersion. Over storage, total carbohydrates increased, while potassium remained at substantial levels until the final day. Weight loss and respiration declined steadily, and sprouting was absent, suggesting extended endodormancy in this genotype. Major deterioration causes observed upon reception included fragmentation, insect damage, and surface molds, highlighting the importance of improved sanitation and mechanical protection during harvest, early postharvest stages, and transportation. Edible coatings enhanced antioxidant activity and increased malic and succinic acid concentrations. Cold storage at 3 °C reduced weight loss more effectively than storage at 12 or 20 °C, although citric acid accumulation was greater at the latter temperature. Among all treatments, immersion at 55 °C for 5 min was the most promising, offering a scalable, low-input option to extend shelf life in neglected yam species.

## 1. Introduction

Edible yam agrobiodiversity holds an untapped potential for the food system’s future, due to these crops’ adaptability to environmental changes, sensory-nutritional value, and phytochemical richness. Colombia ranks among the world’s top yam producers and, notably, is the only non-African country in the top nine [1]. Within Colombia, the Caribbean region stands out for its production volume, genetic diversity, and strong cultural ties to African heritage [2]. However, production is dominated by smallholders: 68% of farmers sell their harvests locally to avoid transport and storage costs, often leading to postharvest issues as weight loss [3].

Among over 30 known edible yam species, South American *D. trifida*, commonly known as yampee or “cush-cush” yam, is the only domesticated American yam still in use. It displays agromorphological traits comparable to those of Asian and African species [4]. Botanically, *D. trifida* is a perennial, dioecious, climbing species of the Dioscoreaceae family, cultivated for its starchy tubers and traditionally propagated by tuber fragments [4]. Several studies have highlighted its nutritional and functional properties, including high carbohydrate and potassium content, as well as bioactive compounds such as anthocyanins, flavonoids, peptides, and polyphenols [5,6,7]. Reported health-related activities include antioxidant and anti-inflammatory effects, and evidence suggests prebiotic functionality [8,9]. However, these traits may vary widely depending on genotype and growing conditions [4,10].

Despite its agronomic potential, *D. trifida* has received limited attention in postharvest research. Available information suggests that the species is prone to rotting, and to a lesser extent, sprouting under high temperature and humidity conditions (30 °C, 76 to 98% RH) [11]. It also has a short dormancy period—about 1 to 2 months—compared to other yams such as *D. alata, D. rotundata,* and *D. dumetorum*, at 3 to 4 months [12]. Although yams are typically susceptible to chilling injury below 10 to 12 °C, *D. trifida* is notable for tolerating storage at 3 °C for up to 30 days [12].

However, early findings indicate that storage outcomes depend on a delicate balance. For example, while cooler temperatures may reduce sprouting and weight loss, they can also increase the risk of rotting [13]. Such studies have also documented sprouting and pest infestations, particularly by mealy bugs, as major causes of deterioration under ambient conditions, especially after one month of storage. These findings highlight the complexity of *D. trifida*’s postharvest physiology and justify the need for carefully balanced interventions to maintain quality.

While detailed physiological studies on *D. trifida* remain scarce, research on related species such as *D. dumetorum* and *D. alata* reveals important biochemical and structural changes during storage, including starch breakdown, lignification, and the accumulation of phenolic compounds as stress responses. These insights help inform the design of postharvest strategies for other underutilized yam species. Several low-cost technologies applied in tropical root crops—particularly sweet potato (*Ipomoea batatas*)—have demonstrated efficacy in improving storage life and product quality. These include hot water dips, in-ground curing, sanitizing, and coatings with honey, aloe vera, or other natural substances [14].

For smallholder producers, such methods are especially appealing as they avoid synthetic inputs and align with agroecological principles increasingly adopted in Colombia.

This study aims to characterize the postharvest behavior of Amerindian purple *D. trifida tubers* at room temperature or refrigeration and evaluate scalable, low-cost GRAS-status technologies such as hot water treatments, calcium treatments with aloe-vera-based coatings to improve storage outcomes of this underutilized yam.

## 2. Materials and Methods

### 2.1. Biological Material and Storage Conditions

Purple *D. trifida* tubers were harvested at commercial maturity (~8 months after planting) from one agroecological farmer in the Montes de Maria subregion (Sucre department, Colombian Caribbean). Tubers were transported under commercial conditions first by road to a distributor in the Bolívar department, and then by plane to Bogotá. They were shipped in cardboard boxes (dimensions: 55 × 55 × 24 cm; ~50 kg each) covered with commercial polyethylene–polypropylene wrapping film. Upon arrival at the analysis facilities of the Universidad Nacional de Colombia, tubers underwent initial sorting to discard damaged or visibly infected individuals. Selected samples were sanitized by wet brushing, immersed in sodium hypochlorite solution (100 ppm) for 5 min, rinsed with water, and dried with forced air at room temperature (20 ± 0.5 °C) for one hour.

### 2.2. Preharvest Microbiological Assessments

As a crucial indicator of tuber survival during storage and a valuable input for future loss prevention strategies—particularly at preharvest and early postharvest stages—microbiological assessments were incorporated into the analysis in a preliminary, exploratory manner through morphological observations. Fungal colonies were observed primarily at the proximal (“head”) and distal (“tail”) ends of the tubers, and occasionally on lateral surfaces with visible injuries or exposed tissue of fragmented individuals.

Microbiological assessment followed the methodology previously reported [14]. In brief, differentiated sections were sampled, cultured on potato dextrose agar (PDA), and incubated for 3 to 7 days at approximately 27 °C, until colonies were developed. Then, macroscopic and microscopic features were evaluated to tentatively identify the main spoilage agents. Microscopic observations were conducted under 10× and 40× objectives using lactophenol blue staining.

### 2.3. Postharvest Study

Reagents used in this study were selected based on their intended applications. Sodium hypochlorite (technical use), calcium chloride, and ascorbic acid (food-grade, for postharvest treatments) were purchased from CIMPA S.A.S. (Bogotá, Colombia). All other analytical-grade reagents used in the physicochemical and compositional analyses were obtained from Sigma-Aldrich (St. Louis, MO, USA), unless otherwise specified.

#### 2.3.1. First Stage: Characterization of the Postharvest Behavior

In the first stage, conditioned tubers were stored at 20 ± 2 °C and 90% relative humidity (RH) for 34 days, following preliminary tests that identified these as optimal conditions for shelf-life assessment [15]. A total of 30 tubers were selected for morphometric characterization, including length, equatorial diameter, and the proximal and distal ends (measured 1 cm from each tip) using a digital caliper (Mahr GmbH, Göttingen, Germany). Tubers were individually weighed and then randomly assigned to two groups: one for destructive analysis and one for non-destructive monitoring. The non-destructive group included at least five tubers per sampling point (*n* = 5), while destructive variables were analyzed in triplicate (*n* = 3) per date. This sampling structure was based on preliminary assessments of variability within the population.

A proximate analysis was conducted at the beginning and end of storage. Non-destructive variables were recorded at 24 time points across the 34-day period, and destructive measurements were taken at eight sampling dates. Physicochemical and functional characteristics were evaluated as described below.

#### 2.3.2. Second Stage: Postharvest Technologies

For the second phase of the study, previously cleaned and disinfected tubers underwent a curing process at 30 °C and 85% RH for 65 h. They were then stored at 20 °C and 90% RH for 96 h to stabilize surface conditions. After this, the tubers were subjected to three independent experiments, each with its own experimental design:

(a) Cold storage was completed under a completely randomized design. Tubers were stored at 3, 12, and 20 ± 0.5 °C (84 ± 6% RH), with three biological replicates per treatment, using independent individuals as analytical units. These temperatures were selected to include a typical ambient condition (20 °C), a moderate low temperature critical to avoid chilling injury for most yams (12 °C), and the recommended refrigeration level for *D. trifida* based on previous reports [12].
(b) Edible coatings were applied following a two-step approach: a calcium chloride (CaCl_2_) dip followed by an *Aloe vera*–sesame oil coating. The experiment followed a 2 × 3 factorial design (Table 1), with three biological replicates per treatment, using independent individuals as analytical units.

In the first step, disinfected and air-dried tubers were immersed in CaCl_2_ solutions (0–2%) combined with 1% ascorbic acid (*w*/*v*) for 1 h, followed by drying at 20 ± 0.5 °C for 1 h. This combination was selected based on its reported effect in reducing enzymatic browning and preserving firmness by inhibiting PPO, POD, PAL, and pectin esterase [16,17].

In the second step, fresh *Aloe vera* gel was prepared according to Qamar et al. [18], including disinfection, extraction, blending, filtration, and pasteurization at 70 ± 2 °C for 40 min. The gel was enriched with 1% ascorbic acid (*w*/*v*) to prevent oxidation, and 0.5% sesame oil (*w*/*v*) to improve hydrophobicity, based on preliminary trials and previous studies [18,19,20,21]. Pretreated tubers were dipped in the *Aloe*–oil blend for 1 min and air-dried at 20 ± 0.5 °C and 90% RH until a uniform coating was formed.

(c) Hydrothermal treatments (HT) were applied by immersing whole tubers in drinking water at temperature–time combinations defined by a central composite rotatable design, 2^2^ + star (Table 2), with three biological replicates per treatment, using independent individuals as analytical units.

The tested range (26–55 °C, 3–17 min) was based on previous studies in *Ipomoea batatas* [22,23] and *Dioscorea* spp. [24], where immersion below 60 °C reduced microbiological spoilage and sprouting without altering sensory or physicochemical traits. Higher temperatures were avoided to prevent starch gelatinization and textural damage.

After treatment, tubers were dried (20 ± 0.5 °C, 80% RH) to prevent mold growth and stored at 20 ± 0.5 °C and 90% RH. All samples were stored for 34 days, with sampling at days 1, 7, 14, and 34 for physicochemical variables, and at days 1 and 34 for starch, sugars, organic acids, antioxidants, and bioactive compounds.

### 2.4. Tuber Characterization

#### 2.4.1. Lyophilization for Phytochemical Analysis

Frozen samples were dried in aluminum trays (116.31 ± 17.62 g per tray, <5 mm depth) using a vacuum freeze-dryer (EV-50 Raypa, Barcelona, Spain) at 45 °C and −0.6 mbar for 21 h. The dried material was ground with a ceramic mortar and stored in sealed pouches for further analysis.

#### 2.4.2. Key Nutritional Components and Metabolites

Proximate composition was determined using AOAC methods: 934.06 for moisture, 900.2 for ash, 920.177 for lipids, 920.176 for protein, 985.35 for magnesium, calcium, potassium, sodium, iron, zinc, and copper contents, 985.29 for total dietary fiber, and 991.43 for soluble and insoluble fiber [25].

Amylose and amylopectin contents were determined following [26] using iodine-based colorimetric reactions. Lyophilized powder (20 mg) was suspended in NaOH and water, vortexed, centrifuged, and filtered. Extracts were reacted with HCl and KI, incubated in the dark for 20 min, and absorbance was measured at 548 nm and 700 nm. Standard curves for both polymers were used for quantification (r^2^ > 0.97).

Sugars (raffinose, sucrose, glucose, fructose, ribose), glycerol, and organic acids (oxalic, citric, malic, succinic, acetic) were extracted from dried samples according to [27] and analyzed by HPLC (Agilent 1200 Infinity, Aminex HPX-87H column, Bio-Rad Laboratories®, Hercules, CA, USA) using 5 mM H_2_SO_4_ as eluent (0.5 mL/min) and RID-VWD detection. Each injection volume was 20 μL in a 20 min isocratic run.

#### 2.4.3. Physicochemical Traits and Antioxidant Analysis

Fresh weight, respiration rate, and surface color were evaluated in non-destructive samples at 1, 2, 4, 7, 9, 11, 14, and 34 d (characterization phase), and 1, 8, 14, and 34 d (postharvest treatments). Weight loss was calculated using Equation (1):

(1) $$W_{loss}=\frac{W_{0}-W_{f}}{W_{0}}\times 100$$

Respiration was measured using calibrated CO_2_ sensors (Vernier, Beaverton, OR, USA) in sealed 200 mL chambers. After 10 min of equilibration, CO_2_ production was recorded for 600 s and the respiration rate calculated using a linear model, adjusted to local conditions, as described by [28], as shown in Equation (2).

(2) $$RI=\left(\frac{3600\times \left(V_{c}-V_{t}\right)\times \rho \times m}{w}\right)\times 0.74$$

where *V_c_:* chamber volume in L*; V_t_:* tuber volume in L; *ρ*: air density at 20 °C in kg·m^3−1^, *m*: respiration rate slope from the LabQuest data fit, in ppm; s^−1^, *w*: tuber weight in kg; 0.74 is the rate of pressure at room temperature, 293.15 K (Bogotá atmospheric pressure, 560 mm Hg, divided by pressure at sea level).

Surface color was recorded at three equatorial points per tuber (CR-400 Chroma Meter Konica Minolta, Ramsey, NJ, USA), under D65 illumination. Color parameters (L*, a*, b*, chroma, hue, ∆E $=\;\sqrt{{∆L^{*}}^{2}+{∆a^{*}}^{2}+{∆b^{*}}^{2}\;}$) were calculated from CIELab* coordinates.

Firmness (N) was assessed using a penetration test with a 2 mm cylindrical probe (LS1, Lloyd Instruments—AMETEK Inc., Berwyn, PA, USA), following the protocol by [29]. Inner color was recorded with the Chroma Meter on three points of exposed tissue, from three individuals per sampling point and treatment.

Moisture content was measured gravimetrically (XM60, Precisa Gravimetrics, Dietikon, Switzerland) on 0.5 ± 0.05 g of tissue dried at 105 °C. Total soluble solids (°Brix) were quantified following a modified version of [30], from juiced supernatants using a digital refractometer (HI 96801, Hanna Instruments, Padova, Italy).

#### 2.4.4. Bioactive Compounds Assessment

Bioactive analysis was adapted from [31,32,33]. First, for anthocyanin extracts, lyophilized powder (2 g) was extracted with 20 mL acidified ethanol (9:1, 70%) for 60 min at 40 °C, and then centrifuged at 4200× *g*, filtered, and re-extracted. Combined extracts were stored at −20 °C until analysis. The extraction method was optimized for *D. trifida* after preliminary comparisons with protocols developed for sweet potato [31], purple maize [33], Asian purple yam [34,35], and a previous study on *D. trifida* [36].

Anthocyanins were determined via the pH-differential method [33]. Extracts were reacted with buffers at pH 1.0 and 4.5, and absorbance was read at 520/700 nm. TAC was expressed as mg C3G/g DW, calculated using Equation (3):

(3) $$TAC=\frac{A\times M_{C3G}\times DF\times V}{e\times L\times W_{ds}}$$

where *A* = (A_520_ − A_700_)_pH 1_ − (A_520_ − A_700_)_pH 4.5_, M_C3G_ = 449.2 Da from Cyanidin-3-glucoside mass; *DF* = dilution factor, *V* = final volume, *e* = Cyanidin-3-glucoside molar absorbance of 26,900 L·mol^−1^·cm^−1^, and *L* = cell path length, 1 cm; and *W_ds_* = weight of dry sample in mg.

Considering evidence highlighting the limitations and complementarity of in vitro antioxidant assays [37], due to the complexity of antioxidant mechanisms, no single assay can fully capture the diversity of antioxidant activity. Three complementary methods were employed—DPPH, TEAC, and FRAP—to reflect both electron transfer and hydrogen atom transfer mechanisms in this study. Reactions were standardized with Trolox and absorbances measured at 517, 734, and 593 nm, respectively, using adapted techniques from [31].

Total phenolic content (TPC) was approached using the Folin–Ciocalteau method [38,39,40], with gallic acid standards. Absorbance was read at 765 nm, and results were expressed in mg GAE/g DW.

### 2.5. Statistical Analysis

All analyses were conducted in R software (v4.4.2) via RStudio (v2023.06.0; Posit Software PBC, Boston, MA, USA). To explore postharvest behavior, a baseline table was created, followed by a Principal Components Analysis (PCA), used to reduce dimensionality and identify key variables. All variables were standardized to a zero mean and unit variance. Profile analysis was applied to significant variables over time, and results were visualized using biplots.

To evaluate treatment effects, repeated-measures multivariate analysis of variance (MANOVA RM) was applied to grouped variables according to data type: non-destructive traits, destructive traits, antioxidants, sugars + glycerol, and organic acids. Univariate tests were conducted for variables with significant interactions (paramBS MATS *p* ≤ 0.05), and only those were retained in refined MANOVA models. Given the data structure, permutation tests and the Wald-type Statistic (perm-WTS) were applied to ensure robust significance estimation (*p* ≤ 0.05).

## 3. Results

The findings from the postharvest evaluation of purple *D. trifida* tubers are presented below, beginning with preharvest defects and quality traits at harvest, followed by changes observed during storage at ambient temperature (20 °C).

### 3.1. Preharvest Defects in Purple D. trifida Tubers

Upon reception, 31.4% *w*/*w* of the tubers were rejected (Supplementary Figure S1) due to three main causes:

• Sprouting (31% *w*/*w*), indicating active growth, accompanied by visible sprouts (Supplementary Figure S1a,b).
• Physical damage (30% *w*/*w*), which increases the risk of accelerated metabolic activity and microbial contamination. Damaged tubers were either broken into multiple pieces or showed clear splitting, typically at the ends, with healed and dry tissues.
• Biological hazard (39% *w*/*w*), which may lead to rapid decay and threaten the safety of the entire lot. Affected tubers exhibited the following:
  - Dry rotting symptoms like discolored or necrotic tissue (Supplementary Figure S1c);
  - Fungal structures, appearing in localized clusters and velvety or cotton-like growths in white, yellow, or green, especially near the proximal end or at visible wounds (Supplementary Figure S1d);
  - Circular holes (1–3 mm diameter) leading to internal tunnels, sometimes containing insect eggs, larvae, or live ants (Supplementary Figure S1e–g).

Visible fungal infections exhibited varied visual features: white cottony tufts or fibrous surface growths, yellow to reddish-brown colonies with pale halos, and grayish-black floccose ranging from compact mounds to loose aerial structures, often with a cottony texture. At least two types of green colonies were noted: rich green sporulating compact mounds of granular texture often associated with distal zones, and blue-green velvety growths with well-defined contours and pale borders (Supplementary Figure S1).

Colony textures in Petri dishes ranged from cottony to velvety and floccose, occasionally furrowed, with surface pigmentation varying from white and green to gray, yellow, and pink to orange hues. Microscopic observations revealed four main distinct morphotypes. One group exhibited brush-like conidiophores (“penicillus” type), ampulliform to elongated phialides, and globose conidia, consistent with *Penicillium* spp. [41]. Another showed canoe-shaped septate macroconidia, matching descriptions of *Fusarium* spp. [42]. And a third type presented broad, hyaline, coenocytic hyphae and dark, rounded sporulating structures, lacking vesicles or phialides; some hyphae appeared to connect through stolon-like bridges, consistent with *Rhizopus* spp. [43]. Similar colonies with green pigmentation for spores, blue for hyphae and conidiophore heads, added to vesicle-like structures were compatible with *Aspergillus*.

While genus-level identification was the limit of this exploratory evaluation, the fungal profile observed aligns with previous reports of common spoilage organisms in *Dioscorea* and other storage roots in tropical regions [44,45,46].

### 3.2. Physicochemical, Quality, and Compositional Traits at Harvest and During Postharvest, at 20 °C

**Figure 1:**
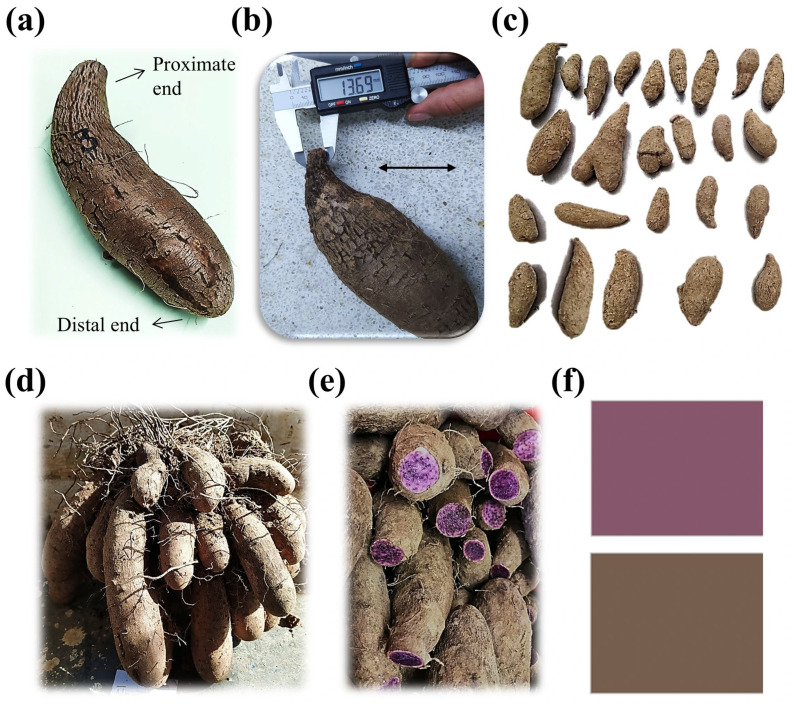
Purple *D. trifida.* (**a**) Healthy tubers, from proximal end (“head”) to distal end (“tail”); (**b**) neck diameter measured using a digital caliper (double arrow lines: 50 mm); (**c**) morphological diversity and size variation; (**d**) fasciculate growth originating shape diversity; (**e**) internal and external coloration; (**f**) simulated color based on average CIELab* coordinates—see Table 2.

Healthy, conditioned yams exhibited a smooth, slightly glossy, silvery-brown thin periderm (Figure 1a), with characteristics comparable to other *Dioscorea* species. Similarities included a cracked surface, but, unlike most yams, purple *D. trifida* yams lacked the typical thick, corky periderm [47]. Tubers were generally fusiform to cylindrical, with morphometric averages of 28.52 ± 3.33 mm at the distal end (“tail”), 13.63 ± 3.18 mm for the proximal end (“head”—Figure 1b), and 45.19 ± 4.98 mm at the equatorial region.

Lobed or irregular shapes were occasionally observed, resulting from the clustered or fasciculate development of these underground storage organs (Figure 1c,d). In a single harvest, a *D trifida* plant may yield up to 25 individual tubers, each 20 to 40 cm in length [48].

None of the defects observed after harvest (Supplementary Figure S1) were evident during storage. Tubers showing early signs of microbiological contamination after 7 d were immediately discarded. These issues highlight species—and location—specific postharvest challenges, which differ from those reported for other *Dioscorea* species and cultivation zones.

Respecting overall characteristics, the average length of the purple *D. trifida* tubers in this study was 151.25 ± 21.82 mm, consistent with previous reports of 15–20 cm for common purple varieties [49]. This value may be lower than those recorded for some of the 25 documented *D. trifida* varieties cultivated under different conditions than those in the Colombian Caribbean, where factors like soil type, mineral nutrition, radiation, irrigation, and genotype influence tuber size.

Tuber weight was highly variable, ranging from 60 g to 360 g across three harvests sampled over two consecutive years from the same region. While Venezuelan accessions have been classified into “purple” and “black” categories based on inner pigmentation intensity [50], this study considered all purple-fleshed yams as a single group, in accordance with local commercialization practices (see Figure 1e color pattern). Colombian average weight fell within the reported range—216.3 ± 35.5 g for “purple” and 112.37 ± 31.66 g for “black” Venezuelan genotypes [5]—and was higher than the 105 g average for Brazilian *D.* *trifida* seedlings [51].

**Figure 2:**
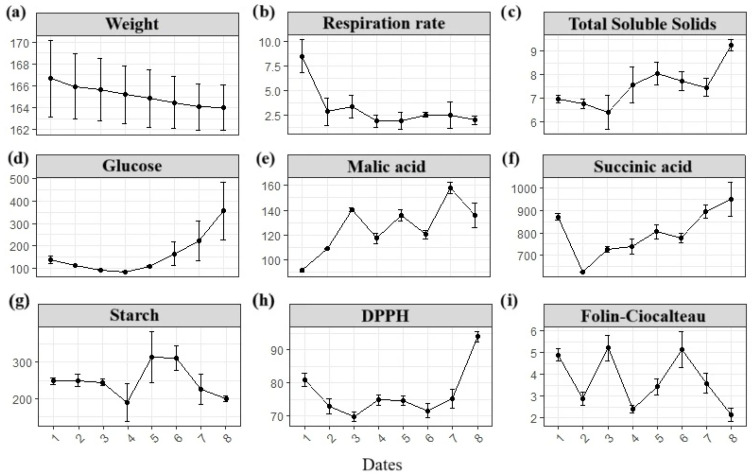
Profile plots of significant variables in *D. trifida* tubers over the 34 days of storage. Measured dates: days 1, 2, 4, 7, 9, 11, 14, and 34. Weight (**a**) is expressed in grams, respiration rate (**b**) in mL CO_2_∙kg^−1^∙h^−1^, and total soluble solids (**c**) in °Brix. Glucose and organic acids (**d**–**f**) are in mg/kg, starch (**g**) in mg/g, DPPH (**h**) in µmol Trolox/g, and Folin–Ciocalteau (**i**) in mg GAE/g DW.

Postharvest losses in purple *D. trifida* were minimized under the tested storage conditions. Similarly to *D. alata* in Costa Rica—where losses are mainly due to nematode injuries, external rotting following harvest, or transportation [52]—*D. trifida* tubers may be susceptible to similar issues. However, the absence of sprouting, unpleasant odors, or visible decay over the 34-day storage period was likely due to the combination of proper sanitation, careful handling, and the tubers’ intrinsic endodormancy. These observations are aligned with the tubers’ respiration rate profile (Figure 2), which suggests Colombian Caribbean *D. trifida* tubers may exhibit a longer dormancy than the Brazilian varieties [51].

While previous studies have noted dormancy periods of about [45] 2 weeks at 28 °C and 80% RH [53], tubers in this study retained acceptable quality over 34 days at 20 °C and 90% RH. This extended dormancy remains shorter than that of other *Dioscorea* species, such as *D. rotundata* or *D. composita,* which can be dormant for three to nine months [53].

Moisture values (Table 3) did not follow the same trend as weight loss at 20 °C (Figure 2).

Although minor fluctuations in moisture content have been reported for some yam species, values generally remain stable during storage [54]. In this study, weight was assessed non-destructively on the same individuals over time, while moisture content was measured destructively on different tubers at each time point. This methodological difference likely explains the apparent discrepancy. Additionally, localized dehydration may occur in the outer tissue without reflecting a measurable reduction in whole tuber moisture content, particularly when tubers remain metabolically active and exhibit water redistribution from inner tissues.

At the end of the storage period, tubers were near the 10% weight loss threshold generally considered acceptable for marketability (Figure 2), with 5% limit being exceeded as early as day 7. This highlights weight loss as a critical factor to consider in the postharvest management of purple *D. trifida*, especially when aiming to minimize quality deterioration and associated economic impacts—whether through pretreatments, improved packaging, or optimized storage conditions. In comparison, storage losses in *D. cayenensis, D. rotundata*, *D. alata*, or *D. esculenta* have been reported to reach 10–15% within the first three months and 30–50% after six months [55]. After one month—similar to the duration of the present study—weight losses of 1–6% have been observed in guinea yams, *D. rotundata* and *D. cayenensis* [56]. The greater loss in purple *D. trifida* indicates a shorter shelf life for this species compared to more commonly cultivated yams.

Storage at 20 °C significantly altered both their external and internal quality parameters of the tubers. Weight and respiration rates stayed within the expected ranges for fresh tubers on arrival (Table 4).

Moderate respiration rates observed at arrival (Table 4) were within the range reported for various *Dioscorea* species, typically between 5 and 25 mL ∙kg^−1^∙h^−1^ of CO_2_ at room temperature (~20 °C) [15,57]. In *D. trifida*, the rates rapidly declined to nearly half their initial value and remained low throughout the 34-day storage period at 20 °C (Figure 2), pointing to the onset of endodormancy. This trend is comparable to that reported for *D. rotundata*, where respiration decreased from 15 to 3 mL ·kg^−1^∙h^−1^ of CO_2_ at 25 °C [57]. The observed reduction in fat content at 20 °C (Table 3) may be linked to its use as a primary respiration substrate and/or its involvement in the biosynthesis of signaling molecules related to stress responses, such as jasmonate, cutin or suberin, or even cytotoxic lipid intermediates derived from membrane remodeling, such as diacylglycerol [58].

External and internal color changes followed different trends. Flesh color, which was more visually heterogeneous (Figure 1e), presented a “mottled” appearance, characteristic of this purple yam genotype [10]. Proximal ends were the darkest, while distal ends were the lightest, lacking the marbled pattern seen in the central flesh. Consequently, all flesh color measurements were taken from transverse sections at the widest equatorial section, to ensure consistency and comparability. Regarding periderm color, the available literature describes only Brazilian *D. trifida* accessions, whose white and purple tubers present darker, greener, and more yellow hues than those measured in this study [59]. This is consistent with their lower L* and a* and higher b* coordinates compared to our results (Table 4). Such color differences may arise from varietal differences in pigment composition—potentially higher carotenoid levels—along with differences in oxidative susceptibility or suberization, both of which can affect postharvest appearance. Flesh color comparisons with Brazilian purple tubers of the same species also revealed a higher L*, and lower a* and b* values, indicating a lighter color with fewer red and blue hues [59]. In comparison with other purple tubers, our results had lower L* values than ten Chinese purple sweet potato varieties (Table 4), which also displayed broader variability in a* and b* coordinates [60]. However, they were comparable to Thai purple *D alata* [61] and Malaysian purple sweet potatoes [62], though the latter showed lower a* values and a tendency toward yellowish hues, possibly reflecting lower anthocyanin content due to varietal and cultivation differences.

Beyond color coordinates, other quality indicators—such as total color change (ΔE), dry matter, firmness, organic acids, and carbohydrate composition—also varied during storage. The periderm ΔE ranged from 1.3 to 14.8, with a mean of 6.1 ± 2.7 after 34 d. Flesh color ΔE averaged 1.99 ± 1.24 (min: 0.6, max: 8.8), indicating perceptible changes to the human eye. Larger changes in periderm color were likely due to exposure to oxygen and manual handling, which can trigger lignification or suberization, and result in surface thickening and darkening [63]. In contrast, Malaysian sweet potatoes stored for only 10 days showed much larger changes in CIELab* coordinates (8%, 29%, and 26%, respectively) [62], while Colombian *D. trifida* showed smaller variations after 34 days (3.7%, 0.8%, and 2.7%, respectively), suggesting a more stable pigment profile in this species.

Dry matter content in purple *D. trifida* (Table 4) was higher than that of *D. alata* tubers stored for 7 to 11 months, which ranged from 23.8 to 29.4% [64], but comparable to the levels reported in fresh Beninese *D. rotundata* and *D. alata* tubers (24.5 to 48.6 g/100 g wb), which showed internal variations from 31.5 to 37.2% from tail to head, along the tuber axis [65]. An increase in dry matter during storage is expected as moisture decreases [66,67].

Postharvest metabolism also involves changing organic acid profiles. Succinic, acetic, and citric acids were the predominant organic acids in purple *D. trifida*, followed by malic and oxalic (Table 4). Nevertheless, their evolution over time did not directly reflect their initial concentrations. Acetic acid became undetectable at the final storage stages, while malic acid showed the greatest variability, followed by oxalic, citric, and succinic acids. These compounds are closely associated with respiration and metabolic activity necessary for tuber maintenance during storage [68]. Given the inconsistent presence of acetic acid and the high variability in firmness, both variables were excluded from the statistical analysis of purple *D. trifida* postharvest behavior at 20 °C.

Regarding carbohydrates, sucrose was the predominant sugar in purple *D. trifida* tubers, accounting for approximately 79% of total sugars, followed by smaller amounts of fructose and glucose (Table 4). This sugar profile is in accordance with those of sweet potatoes [69] and other *Dioscorea* species, including *D. rotundata*, *D. alata,* and *D. cayenensis* [70]. Carbohydrate levels tend to be relatively stable during storage, with *D. rotundata* showing decreases of about 6% after one month [71], as observed for this study (Table 3). Conversely, starch content decreased by nearly 20% (Figure 2), probably due to its role as the main storage polysaccharide in vegetable tissues, gradually hydrolyzed during storage to fuel respiration. This process is also influenced by the tissue structure, the biological material’s origin, and genotype.

Raffinose content ranged from 0 to 401.8 mg/kg across all samples and storage times (Table 4), indicating a highly variable presence and a likely minimal contribution to health benefits from purple *D. trifida* consumption, in either raw or dry forms (as was processed for analysis in this study). Similarly, ribose was detected only in trace amounts (0.26 ± 0.57 mg/kg), and given its low dietary relevance and analytical significance, it was not further explored. While some *Dioscorea* tubers have shown increasing concentrations of rhamnose, galactose, arabinose, and maltose after two months of storage—presumably due to starch degradation [70]—these sugars were not predominant in our samples.

Amylose represented 10.6% of the total starch content at the initial sampling point (Table 4), and decreased slightly to 9.4% after 34 days of storage at 20 °C. These values contrast with previous findings that reported a waxy starch in *D. trifida,* with amylose content as low as 3.7% [72] or even below 2% [5]. Our results indicate that Colombian purple *D. trifida* starch may be considered low amylose, but not formally waxy, in agreement with recent reports from southeastern Colombia, with variable amylose levels between 5.7 and 11.2%, depending on extraction methods [26].

As for antioxidant capacity, purple *D. trifida* presented values from 12.1 to 82.9 µmol Trolox/g, as determined by various analytical methods (Table 4), reflecting a noteworthy functional potential linked to their anthocyanins, phenolics, and other antioxidant compounds. These values were comparable to those reported for other edible *Dioscorea* tubers, including *D. trifida*. Total anthocyanin content (TAC) in purple *D. trifida* tubers was comparable to initial anthocyanin reports of this species [36], though lower than recent findings on other species, or in *D. trifida,* obtained through enhanced extraction techniques [73,74]. Total phenolic content also fell below values reported for other purple yam cultivars, particularly in their peel [75], yet remained within the range for other yams. In contrast, DPPH values exceeded those of *D. opposita*, and TEAC was within the range observed in *D. trifida,* depending on drying treatments [73]. While extraction efficiency may influence the absolute concentrations, the presence of a diverse array of amino acids, phenolics, and anthocyanins recently identified in this species [7] underscores its functional relevance. These findings reinforce the potential of Colombian *D. trifida* tubers as a promising dietary source of bioactive compounds, even under standard extraction conditions.

Over the 34-day storage period, three out of four assays (excluding DPPH) showed a decline (See Figure 2). DPPH and FRAP decreased by approximately 15%, while TEAC was reduced by 36%. Both total phenolics and anthocyanins dropped by more than half, reflecting compositional changes associated with postharvest metabolism.

Principal Components Analysis (PCA) of the thirty-four destructive and non-destructible variables revealed two main components accounting for the largest portion of the total variance (20.6 and 16.4%; Supplementary Figure S2). Twenty-four variables showed potential intercorrelations, yet scatterplots and biplots did not reveal distinct groupings among the samples. Examination of the variable contributions to the first cluster identified sixteen variables with above-average influence (Supplementary Figure S3), highlighting their relevance in explaining postharvest changes in purple yampees.

To refine the analysis and minimize redundancy, a correlation matrix was constructed (Supplementary Figure S3). Strong associations were found between starch and its components—amylose and amylopectin, glucose and fructose, and malic acid and oxalic acid. Given that glucose had a higher contribution to the PC1 than fructose, and holds a greater metabolic significance, it was retained for further analysis. Starch was also prioritized over its highly correlated fractions due to its overall nutritional relevance. Oxalic acid, often regarded as an antinutrient, was present at low concentrations (Table 4), well below the 50 mg/100 g fresh weight threshold considered a health concern [76]. It was therefore not deemed a limiting factor for consumption or safety in purple yampee tubers.

### 3.3. Effects of Postharvest Technologies on Tuber Characteristics Along Storage

Based on the postharvest behavior observed in purple yampees and the feasibility for local farmers, three context-appropriate postharvest treatments were selected. Their effects on tuber quality and composition throughout the storage period are described below.

#### 3.3.1. Cold Storage

**Figure 3:**
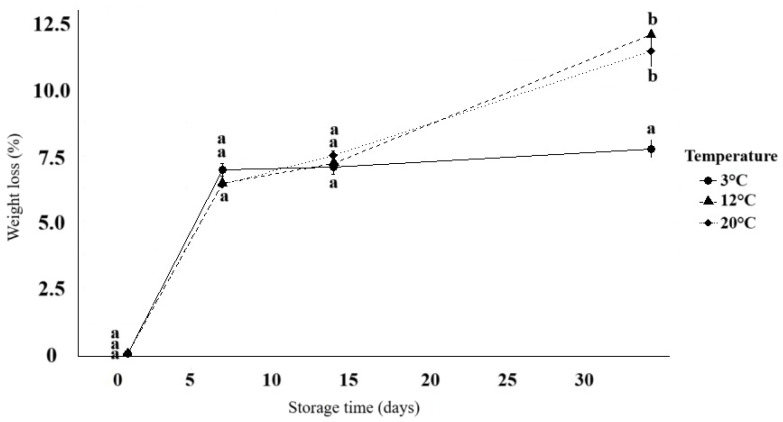
Profile plot of weight loss (%) in *D. trifida* tubers stored at 3 °C (●, solid line), 12 °C (▲, dashed line), and 20 °C (◆, dotted line) over 34 days of storage (days 1, 7, 14, and 34, mean ± SD, *n* = 3). Different letters indicate significant differences according to WTS (*p* = 0.05).

**Figure 4:**
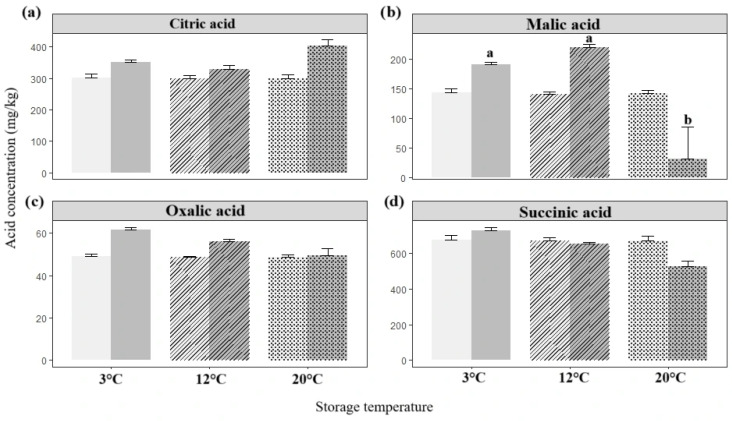
Changes in (**a**) citric, (**b**) malic, (**c**) oxalic, and (**d**) succinic acid concentrations (mg/kg, mean ± SD) in *D. trifida* tubers at the beginning (day 1, light gray) and end (day 34, dark gray) of storage at 3 °C (solid bars), 12 °C (striped bars), and 20 °C (dotted bars). Error bars represent standard deviation (*n* = 3). Significant differences (malic acid only, *p* < 0.05) were identified using MANOVA RM (paramBS (MATS) and perm-WTS.

Storage temperature had a limited impact across most variables, except for weight loss and organic acid concentrations (Figure 3 and Figure 4). Multivariate analysis revealed no significant interactions between time and temperature for sugar content or physicochemical traits (Supplementary Table S1). In contrast, perm-WTS confirmed significant differences for weight and organic acids (*p* < 0.05).

Weight loss profiles differed significantly by temperature (*p* < 0.001 for flatness and parallelism; *p* = 0.011 for level differences; Figure 3). Losses were lower at 3 °C after day 7, reaching 7.66% by day 34, compared to 12.01% at 12 °C and 11.24% at 20 °C.

Organic acid levels were mostly stable, except for a significant decline in malic acid at 20 °C (Figure 4). Citric acid concentrations increased by 34.4% at 20 °C, 16.2% at 3 °C, and approximately 10% at 12 °C.

Cold-induced physiological injuries have been reported in yams stored below 10–12 °C, including altered respiration and physical degradation [77]. For example, *D. rotundata* presents chilling injury at 12.5 °C, while *D. alata* experiences tissue breakdown within 3 to 4 weeks at 3 or 12 °C [78]. Although no prior studies have reported on cold storage physicochemical or physiological responses in *D trifida,* its tolerance may resemble that of purple sweet potatoes (*I. batatas)*. These tubers maintained starch, anthocyanin, and phenolic content and limited weight loss when stored at 4–5 °C for up to 6 months, although storage at 5 °C might relate to cold-induced sweetening in some cases [79,80,81]. In this study, cold treatments had no significant effects on sugar increasing, respiration rates, or alcohol content (Supplementary Figure S4).

Glycerol is known to participate in responses to metabolic stress [82], and raffinose has been described as a cold-stress marker in various tissues, including beet roots [83,84]. In the present study, however, neither glycerol nor raffinose levels differed significantly over the 34-day storage (mean ± SD, *n* = 3; Supplementary Figure S5), suggesting that storage at 3 or 12 °C did not trigger cold stress responses in purple yampee tubers.

#### 3.3.2. Coating Treatments

The calcium dips combined with aloe-based coating had a limited effect on only six quality parameters of purple yampees during postharvest storage, according to a preliminary MANOVA (Supplementary Table S1). Variables showing no significant treatment x time interactions in univariate analysis (*p* > 0.05) included firmness, dry matter, TSS, internal color coordinates (CIELab*), oxalic acid, starch, amylose, amylopectin, glucose, fructose, ribose, glycerol, and FRAP assay results. A refined MANOVA RM excluding these variables confirmed no significant effects for destructive traits (paramBS MATS = 0.091), but revealed interactions for the non-destructive, organic acids, sugars, and antioxidants groups (paramBS MATS = 0.003, 0.006, 0.001, and 0.013, respectively; Supplementary Table S1). Nonetheless, among non-destructive traits, no time x treatment interactions emerged in subsequent univariate analysis.

**Figure 5:**
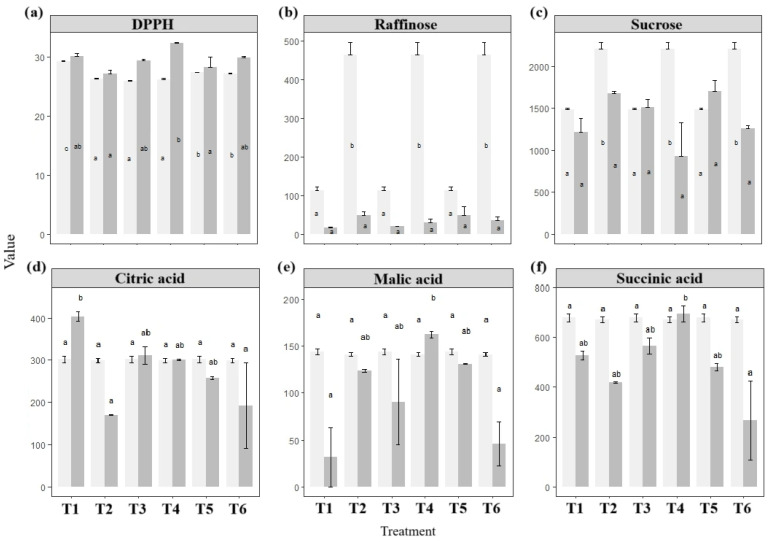
Evolution (mean ± SD, *n* = 3) of (**a**) antioxidant capacity (DPPH, µmol Trolox/g), (**b**) raffinose (mg/kg), (**c**) sucrose (mg/kg), and (**d**–**f**) citric, malic, and succinic acids (all in mg/kg) in purple *D. trifida* tubers subjected to calcium pretreatments, and *A. vera*-based coatings on days 1 and 34 of storage. Treatments: T1, Control; T2, CaCl_2_ (2%); T3, CaCl_2_ (1%); T4, CaCl_2_ (2%) + aloe; T5, aloe; T6, CaCl_2_ (1%) + aloe. Different letters indicate significant differences according to perm-WTS (*p* < 0.05).

As illustrated in Figure 5, significant differences (*p* < 0.05) were observed in DPPH antioxidant capacity, as well as in sucrose and raffinose among sugars, and citric, malic, and succinic among the organic acids. DPPH values were lower than those measured during initial tuber characterization for most treatments containing CaCl_2_, ranging from 26.03 to 32.40 µmol Trolox/g. The greatest relative increase in antioxidant capacity was recorded in T4 (23.55%), followed by T3 (12.92%) and T6 (9.73%), while changes in the remaining treatments were below 3% compared to their initial values.

Among the sugars, raffinose exhibited the greatest variation in T2, T4, and T6. This variation may have been influenced by higher initial sugar levels in these treatments. These were also the treatments in which sucrose degradation was least pronounced: 57.75% for T4, 42.83% for T6, and 24.06% for T2. As all three included CaCl_2_ (1–2%), these findings suggest a potential role of calcium in preserving sucrose during yampee storage.

Citric acid showed the most pronounced changes in the untreated control (T1), while remaining relatively stable in T3, T4, and T5, in contrast to T2 and T6 (Figure 5).

#### 3.3.3. Hydrothermal Treatments

Among all evaluated technologies, hydrothermal treatments had the most pronounced impact on the postharvest quality of purple yampees. Statistical analysis (*p* < 0.05) revealed significant effects in 16 out of the 34 variables assessed. Following repeated measures MANOVA, all variable groups except for non-destructive traits (paramBS MATS = 0.849) exhibited significant time x treatment interactions.

**Figure 6:**
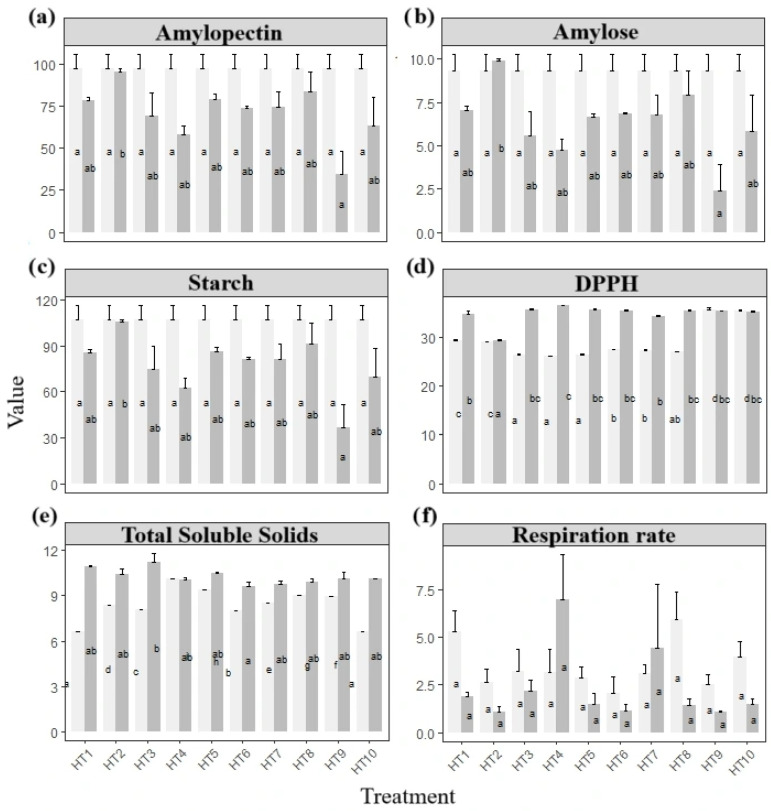
Evolution (mean ± SD; *n* = 3) of (**a**) amylopectin, (**b**) amylose, (**c**) total starch (all in mg/g DW), (**d**) antioxidant capacity (DPPH, µmol Trolox/g), (**e**) total soluble solids (°Brix), and (**f**) respiration rate (mL CO_2_·kg^−1^·h^−1^) in *D. trifida* tubers after hydrothermal treatments, at day 1 (light gray) and day 34 (dark gray) of storage at 20 °C and 90% RH. Hydrothermal treatments: HT1, 20 °C—10 min; HT2, 26 °C—5 min; HT3, 26 °C—15 min; HT4, 40.5 °C—3 min; HT5, 40.5 °C—10 min; HT6, 40.5 °C—10 min; HT7, 40.5 °C—17 min; HT8, 55 °C—5 min; HT9, 55 °C—15 min; HT10, 61 °C—10 min. Different letters indicate significant differences according to perm-WTS (*p* < 0.05).

Univariate analysis further confirmed that respiration rate was significantly influenced by HT (perm-WTS = 0.001), with most treatments showing a reduction over storage, except for HT3 and HT6 (Figure 6). This outcome reflects the physiological sensitivity of yampees to specific hydrothermal conditions and their effect on metabolic activity.

Principal Components Analysis identified starch, amylose, amylopectin, TSS, and DPPH as the main contributors to PC1 (Supplementary Figure S4), and these were selected for further analysis based on their influence on data structure. Optimization analyses were performed to identify the conditions minimizing respiration rate (a key physiological trigger for most traits) and weight (a critical determinant of marketability). However, surface response models did not yield significant results, with *p*-values of 0.086 for weight loss and 0.622 for respiration rate. Optimization for amylose, starch, and amylopectin yielded a desirability score of 89.57% at 61 °C for 17 min, but non-significant *p*-values (0.89, 0.93, and 0.9315, respectively) indicated inconclusive effects for these variables.

Although no statistically significant differences were detected among the treatments over storage (*p* > 0.05), overall weight loss remained at acceptable levels for most hydrothermally treated tubers. After 1 week at 20 °C, the mean weight loss was 2.1 ± 0.3%, increasing to 7.1 ± 0.9% by day 34. The only exception was HT3, which exhibited the highest final weight loss of 10.2%.

## 4. Discussion

Based on the findings, several important points arise that merit discussion, starting with the tubers’ condition upon arrival. The visual characterization of defects—including sprouting, injuries, and microbial decay—revealed critical challenges in preharvest and early postharvest practices. A considerable number of losses had already occurred before the start of the storage trial, due to a combination of mechanical damage, pest activity, and microbial spoilage, as illustrated in the Supplementary Figure S1. These observations helped contextualize the subsequent deterioration patterns and underscore the need to improve quality control from the earliest stages of the supply chain.

The preliminary identification of fungal genera associated with visible decay contributes to a broader understanding of the biological deterioration in *D. trifida*. The dominant groups—*Fusarium, Penicillium, Aspergillus*, and *Rhizopus*—have been reported in stored root and tuber crops, including sweet potato and white- and purple-fleshed yams [41,43,44,45,46,85,86]. According to such references, these fungi thrive at room temperature and can be introduced through soil, crop residues, or environmental exposure, and frequently infect storage roots via wounds sustained during harvest or transit.

In our study, most infections were concentrated at the proximal (“head”) and distal (“tail”) ends of the tubers, where detachment from the plant and surface abrasions typically occur. This spatial pattern strongly suggests that field practices—including extraction, handling, and packing—may represent the first critical window of infection. Recent studies confirm this pathway: *Fusarium* spp. are recognized as soil-borne fungi capable of persisting across seasons and colonizing wounds on storage roots [42,43]. *Penicillium* and *Aspergillus* species have also been isolated from soil, plant tissues, and postharvest environments in both tropical and temperate systems [41,45].

Given these results, it becomes essential to reinforce good agricultural practices in the field, as well as handling storage and transportation stages. Cross-contamination may occur at harvest if cutting tools, digging instruments, or packaging materials are reused across plots or crops without proper disinfection, allowing soilborne pathogens to penetrate exposed tissues—especially at the proximal end of the tubers, where a first injury takes place for tubers individually. Strengthening cleaning protocols for harvesting equipment and contact surfaces could significantly reduce initial microbial ingress. Beyond the field, mechanical damage during handling and transportation must also be minimized. Tubers should be cushioned to prevent compression and abrasion, and packaging can incorporate physical separators or soft-fill materials to limit friction and bruising. Additionally, encouraging farmers to harvest and distribute tubers in their natural growth fascicles (Figure 1d) could help protect the proximal zone from exposure and reduce contamination risk during large-scale distribution. These measures are especially relevant given that, in Colombian yam supply chains, the same fungal genera identified here (*Fusarium*, *Penicillium*, *Aspergillus*, and *Rhizopus*) have been reported as key contributors to storage losses [44], and mechanical injuries alone have been associated with losses of 14–17% in other *Dioscorea* tubers from the Caribbean [46].

Although our identification was morphological and exploratory, the deterioration patterns observed reveal a critical vulnerability that warrants further research using molecular techniques and long-term storage trials.

### 4.1. Postharvest Behavior and Biochemicals Evolution at Ambient Conditions (20 °C)

Six compositional traits in purple yampees declined to varying degrees over 33 days of storage, with the most notable reductions observed in crude fat (−79%), calcium (−28%), and magnesium (−15%) (Table 3). Similar trends have been reported in *D. cayenensis rotundata* and *D. alata*, where these components either decrease or remain stable during storage [66,67]. In *D trifida,* the contents of sodium, iron, and potassium also decreased markedly (Table 3). As these minerals are involved in gene regulation, energy generation, and oxygen transport [87], their decline likely reflects ongoing metabolic activity.

In contrast, carbohydrate content showed moderate increases over time (Table 3), consistent with previous reports in yams, where polysaccharides are enzymatically converted into simple sugars or organic acids during storage [12,66,67,88].

Compared to purple *D. trifida* tubers grown in Venezuela [5], the Colombian samples exhibited lower moisture content but higher levels of crude protein, fat, and ash. Carbohydrate levels were greater in the Venezuelan accessions, while dietary fiber was comparable. Notably, the Colombian yams displayed a higher proportion of insoluble fiber (89.3%) than previously reported. Regarding mineral composition, calcium and magnesium contents exceeded the 40 mg/100 amounts from Venezuelan accessions, while sodium and iron were in a similar range [5,89]. These results suggest that Colombian Caribbean *D. trifida* may serve as a robust source of key minerals, particularly Mg, Zn, and Ca, with higher storage stability potentially influenced by soil and cultivation practices.

Color stability emerged as a relevant trait in this study, especially considering the perceptible changes registered during storage. The flesh color of *D. trifida* tubers showed a moderate total color change (mean ΔE ≈ 2 units), with smaller shifts than those observed in the periderm, as expected due to its greater exposure to handling and oxygenation. These results suggest greater pigment stability in the inner tissues of this species.

Although some studies have reported initial color coordinates in fresh purple yam and sweet potato varieties [59,60,61,62], few have tracked postharvest changes in CIELab* coordinates. Among the available literature, only a study on the Malaysian sweet potatoes was found to be comparable, as Nurfarhana et al. [62] assessed the color evolution of the tubers under room temperature storage. Despite a much shorter storage time (10 days), the sweet potatoes’ changes in the L*, a*, and b* coordinates were substantially higher than those of *D. trifida*. A greater stability in the Colombian tuber’s pigments at room temperature may be attributed to differences in anthocyanin composition, vacuolar stability, or more robust antioxidant systems. These comparisons merit further attention, not only for their value in benchmarking pigment stability under tropical storage conditions, but also because postharvest color data on underutilized yam species remains scarce.

Dry matter fluctuated by 9.89% from baseline values. Although this deviated from the expected increasing trend during storage, it may reflect sampling variability or mechanical damage sustained by the tubers used for early destructive analysis. Such damage could have enhanced moisture loss and led to higher solids concentration. The absence of a typical moisture-to-dry-matter correlation underscores the need for greater uniformity in sample selection at the start of postharvest trials, or for establishing standardized grading criteria, to minimize the impact of internal biological variability and sampling effects.

Firmness ranged from 25.3 to 26.9 N (Table 4), in agreement with values reported for *D. alata* [64]. A gradual increase was observed during storage, possibly related to changes in structural carbohydrates (see Figure 2). Previous studies have shown that texture reinforcement occurs within the first 72 h of storage, as starch content degrades [90], indicating that firmness development may result from internal polysaccharide rearrangements such as partial retrogradation or starch redistribution.

Among the organic acids, malic and oxalic acid showed the greatest variation, indicating high metabolic responsiveness. Acetic acid, in contrast, diminished sharply, reinforcing its exclusion from trend analysis. Importantly, oxalic acid concentrations remained low throughout storage (Table 4), aligning with other reports of low oxalate accumulation in other *Dioscorea* species [91] and supporting the classification of purple *D. trifida* tubers as safe food with minimal antinutritional risk.

The observed starch hydrolysis confirms the progressive mobilization of reserves to support respiration and energy metabolism. This contributes to the gradual reduction in physiological and nutritional quality, emphasizing the need for timely consumption or preservation strategies.

Whilst raffinose is recognized as a prebiotic with potential benefits on colonic flora, its concentrations in purple *D. trifida* were insufficient to meet dietary fiber or prebiotic recommendations. No specific threshold has been established for raffinose, but fructooligosaccharides (FOSs) intakes of ~0.8 g/kg are considered acceptable [92]. Only trace levels of ribose were detected, confirming limited nutritional relevance. In contrast, increases in rhamnose, galactose, arabinose, and maltose likely reflect active enzymatic starch degradation, supporting conversion of complex carbohydrates into simple sugars during storage.

The modest decline in amylose content over time may indicate metabolic utilization or partial structural rearrangement of linear starch fractions during storage. This low amylose profile is consistent with previous reports on *D. trifida* [5,72]; however, more recent findings [26] support our hypothesis that purple *D. trifida* tubers from Colombia exhibit a starch composition that does not fully match the characteristics of a waxy phenotype. Such variability may reflect methodological differences or tuber growing conditions.

Amylose synthesis in starch is determined by the GBSSI (Granule-bound Starch Synthase I) gene, known as the “waxy” gene [93], and high-temperature stress has been shown to modulate its expression in maize [94]. Similar environmental stressors, including drought-induced heat stress reported by local firmest during the delayed harvest period, could have contributed to the observed amylose levels in these tubers. Additionally, regional differences in waxy allele expression may partly explain the moderate amylose content observed in Colombian genotypes. From a functional standpoint, low amylose starches are generally less prone to retrogradation and enzymatic hydrolysis, which could enhance their postharvest stability. In this study, a slight average decrease of 1.2% was observed, indicating subtle, yet measurable changes in starch composition during storage.

TEAC values were lower than those reported for *D. alata* and *D. caucasica,* but exceeded those of *D. bulbifera,* a trend mirrored in DPPH results, which were lower than in *D. caucasica* and *D. opposita* [6]. Over the 34-day storage at 20 °C, three out of four antioxidant determinations (excluding DPPH) decreased (see Figure 2). Total phenolic content, measured by the Folin–Ciocalteau method—known to decline in *D. cayenensis* and *D. alata* during storage [66]—remained comparatively high and exceeded values reported for *D. rotundata*, *D. japonica*, *D. versicolor*, *D. deltoidea*, *D. triphylla*, and *D. dumetorum* and some *D. hirtiflora*, *D. bulbifera,* and *D. alata* [6]. Earlier studies report lower phenolic levels in *D. trifida* tubers [36], and variations in extraction protocols—including recent optimization techniques [73]—may explain discrepancies. Likewise, total anthocyanin content surpassed previously reported values for *D. trifida* [36] but remained below those obtained using enhanced methods [26,73] or in other purple yams [74]. These observations reinforce the functional food potential of *D. trifida* tubers, while emphasizing the influence of methodological factors when assessing antioxidant profiles.

Principal Components Analysis identified a subset of chemical and physical traits as key indicators of postharvest responses in purple yampees. Sixteen variables were identified for their high contribution, supporting their relevance in describing storage-induced changes. The lack of consistent grouping patterns among samples further illustrates the phenotypic heterogeneity typical of underutilized crops outside standardized monoculture systems and highlights the need for further investigation into their postharvest physiology.

The selection of glucose and starch for focused analysis was based on both their compositional importance and physiological roles. Glucose serves as a central substrate in respiration, and its concentration directly influences postharvest senescence and decay. Similarly, total starch content offered a more integrative indicator of tuber behavior over time than its two constituent polysaccharides, amylopectin and amylose, which were highly correlated.

### 4.2. Limitations and Benefits of Cold Storage in Neglected Yam Species

In storage organs like yam tubers, as in most fresh produce, cold temperatures slow down enzymatic activity, reduce membrane fluidity, and suppress respiration rates—mechanisms that help delay senescence and reduce metabolic losses. This general response has been widely observed in tropical crops, where moderately low temperatures inhibit degradative pathways and limit the oxidation of key substrates such as organic acids and sugars [29,95,96].

The storage behavior of *D. trifida* at 3 °C may be contextualized within previous findings showing that low-temperature storage effectively reduces weight loss in yams. For instance, Simões et al. [96] demonstrated that storage at 5 °C minimized weight loss even in minimally processed *Dioscorea* spp. This aligns with our observations, where weight loss in purple *D. trifida* tubers was significantly reduced at 3 °C, particularly after the second week. Such results challenge the general recommendation of storing yams at 12–16 °C [56] and indicate that certain species may benefit from lower temperatures.

The benefits observed are also in line with the literature reporting reduced transpiration, metabolic activity, and respiration rates at 5 °C in comparable storage organs, such as sweet potatoes [29] and fresh-cut *D. alata* yams [95]. In the latter, cold storage was also associated with reduced microbial proliferation, particularly psychrotrophic bacteria.

Since metabolic pathways are typically enhanced at higher temperatures, our results (Figure 4) confirm that respiration, particularly via malate intermediates, was more active at 20 °C, while refrigeration slowed cellular activity. The significant reduction in malic acid at 20 °C aligns with prior evidence that malate levels tend to decline in fresh produce during storage [97]. In contrast, cold-stored purple yampees preserved or slightly increased organic acid concentrations, consistent with reduced respiration and slower substrate oxidation. In a comparable case, Ghanaian *D. dumetorum* tubers showed rapid alterations in chemical, biochemical, and textural properties following 24–72 h of exposure to 4 °C [90].

The low raffinose levels observed at 20 °C compared to those stored at 3 and 12 °C (Supplementary Figure S5) may reflect a subtle stress response. Raffinose is known to accumulate in storage tissues under cold stress as a protective mechanism [98]. However, in this study, raffinose concentrations remained largely stable across both refrigeration treatments. The absence of statistically significant differences (*p* < 0.05), coupled with stable external appearance and composition, suggests that storage at 3 and 12 °C did not induce measurable physiological stress in purple *D. trifida* tubers. These results support a degree of low-temperature tolerance in *D. trifida*, at least under the tested conditions.

Taken together, these findings indicate that cold storage at 3 °C modulates metabolic activity in tropical tubers and may confer postharvest benefits to *D. trifida* by reducing physiological stress and slowing nutrient degradation.

### 4.3. Coatings and Calcium: Enhanced Responses

The application of coatings produced limited overall effects, but selected treatments significantly influenced specific traits. Noticeably, antioxidant levels—as measured by the DPPH assay—were higher in T4, T3, and T6 compared to the untreated control (T1). These treatments, which included 1–2% CaCl_2_ with or without *Aloe vera* coatings, may have helped preserve endogenous antioxidants, mitigated oxidative degradation, or promote bioactive compounds production through antioxidant or protective barrier mechanisms.

Although studies on such coatings in yams are limited, similar effects have been documented in other crops. For instance, DPPH radical scavenging activity was retained in cold-stored chillies coated with *A. vera* [99]. In mango, combining chitosan and calcium improved the efficacy of *Aloe vera* coatings, controlling weight loss and antioxidant status, as evidenced by DPPH values [100]. In cassava, bio-emulsion coatings with calcium chloride minimized both weight loss and enzymatic deterioration during storage [17]. These outcomes may reflect the combined antioxidant and antimicrobial action of *A. vera*, which is increasingly recognized as an edible coating for fresh and minimally processed produce [101]. *A. vera* gel contains α-tocopherol, carotenoids, ascorbic acid, flavonoids, tannins, and anthraquinones such as aloe-emodin, which contribute to its radical scavenging activity among other functional properties [101,102].

Calcium chloride is known to stabilize cell membranes and delay senescence by maintaining wall integrity and inhibiting degrading enzymes [103]. In fruits, calcium has also been reported to reduce respiration, preserve organic acids, and minimize the conversion of starches into simple sugars during ripening [104].

The synergistic action between *A. vera* coatings and calcium ions may help preserve biochemical stability in *D. trifida* tubers, as observed in T4 (2% CaCl_2_ + *A. vera*), which showed improved antioxidant activity and organic acid retention during storage.

The maintenance of membrane integrity in coated and calcium-treated tubers may help limit respiration [105], reducing the oxidation of substrates such as sucrose, malic, citric, and succinic acid [106]. These treatments may also prevent excessive water loss and dampen metabolic stress signaling, including the accumulation of raffinose, often associated with osmoregulation and reactive oxygen species (ROS) detoxification [107]. While further research in D. trifida is warranted to validate these pathways, our results support a potential synergistic role of calcium and A. vera in enhancing postharvest quality and biochemical stability in underutilized roots.

### 4.4. Hydrothermal Treatment as a Promising Strategy for Quality Preservation

Hydrothermal treatment significantly affected 16 out of 34 evaluated variables, making it the most effective strategy among those tested. High metabolic activity, particularly respiration, is widely recognized as a main contributor to postharvest deterioration in yams [57]. Conversely, HT is commonly associated with delaying senescence and suppressing respiration [22], as observed in our experiment, both at the beginning and after 34 days, with the exception of HT4 and HT7 (40.5 °C, for 3 and 17 min, respectively; Figure 6).

The temperature–time combinations applied in several HTs (55 °C and 61 °C) fall within the range known to induce protein denaturation [108]. This may impair enzymatic systems responsible for respiration and related catabolic processes, explaining the significant physiological effects observed.

Endogenous enzymes such as peroxidase (POD), lipoxygenase (LOX), and polyphenol oxidase (PPO)—key players in quality degradation—are thermally inactivated above 50 °C. Their half-lives at 50–60 °C range between approximately 2.5 and 4.0 min, depending on the enzyme [109], supporting the efficacy of high-temperature HT in limiting enzymatic degradation.

Treatments above 50 °C may therefore be advantageous to minimize microbiological and enzymatic spoilage, inhibit sprouting, and extend shelf life.

Among all tested technologies, HT resulted in the lowest weight losses after 34 days, highlighting its potential economic benefits for smallholder farmers given its simplicity and minimal training requirements.

Studies on sweet potatoes have shown that HT at 45–50 °C for 10 min reduces softening, delays total soluble solids (TSS) accumulation, and extends shelf life [22]. In yams, although postharvest HT studies remain limited, treatment at 50–53 °C for 20 min has been shown to reduce sprouting, with cultivar-dependent sensitivity [24]. Lower-temperature HT (<50 °C), on the other hand, may trigger physiological changes such as sprouting in sweet potatoes, while treatments at 53–56 °C for <10 min have been shown to minimize spoilage and sprouting [23].

Among our treatments, HT4 (40.5 °C, 3 min) elicited the highest DPPH antioxidant response. Combined with its elevated respiration rate, this suggests a mild heat-induced stress triggering antioxidant defense. In general, heat stress stimulated antioxidant activity across most treatments, particularly from 26 °C (15 min) to 55 °C (5 min), with values maintained in HT9 and HT10.

Treatments such as HT8 (55 °C, 5 min) may offer an optimal balance: enough thermal input to induce protein denaturation while minimizing exposure time, potentially reducing biochemical degradation and energy requirements. This approach likely limits enzymatic oxidative metabolism in the tubers, contributing to improved quality retention.

HT10 (61 °C, 10 min) appeared particularly promising for deeper physiological inactivation, combining reduced respiration rates with high antioxidant retention. However, its greater energy demand may limit adoption by small-scale producers.

Therefore, while high-temperature HTs may offer greater preservation benefits, they should be selected based on the tubers’ intended use and the resources available, balancing effectiveness and cost-efficiency showed promise for further metabolic inactivation, combining reduced respiration rates and antioxidant retention.

However, selecting hydrothermal treatments should take into account both physiological responses and practical feasibility, depending on the intended end use. Based on this, Table 5 proposes exploratory associations between specific HTs and functional postharvest goals in purple *D. trifida* tubers.

### 4.5. General Implications for Yam Postharvest Technology

Among all postharvest treatments performed on purple yampees, citric acid emerged as one of the most sensitive metabolic indicators, showing consistent differences across storage conditions. At 20 °C, its concentrations increased more markedly than at 3 °C or 12 °C. Within the calcium and coating trials, only the untreated control (T1) followed this trend (Figure 5), while combinations involving 2% or 1% CaCl_2_ with *Aloe vera* coating preserved or slightly enhanced citric acid content.

Beyond this response, the synergistic application of calcium dips and *Aloe vera*-based edible coatings showed potential to retain antioxidant capacity and stabilize soluble sugars such as sucrose and raffinose. These effects suggest a protective role against compositional degradation and oxidative stress under ambient storage, thus contributing to shelf-life extension while preserving nutritional and functional quality. These outcomes support the viability of calcium-based pretreatments, especially in combination with *Aloe vera*, as practical options for maintaining biochemical quality under non-refrigerated conditions.

Considering the limited research available on *D. trifida*, further investigation into the species’ physiological responses to postharvest treatments is warranted, particularly within frameworks aimed at sustainable food systems and the valorization of neglected crops.

In parallel, hydrothermal treatment induced measurable changes in both metabolic and physical parameters. Half of the protocols led to increases in citric acid over storage, which—given its central role in the Krebs cycle—could reflect metabolic adjustments to storage-related stressors. Although the sensory or technological implications of these changes remain to be fully explored, their reproducibility across treatments warrants further investigation.

From a practical standpoint, HT emerged as the most promising technology tested, not only for its technical effectiveness in reducing respiration and weight loss, but also for its low-cost implementation potential. These differentiated effects provide a basis for treatment selection according to intended use, responding directly to reviewer recommendations for specific application guidance.

Still, broader adoption of HT will require prior improvements in *D. trifida* production systems, including the availability of pest- and disease-free planting material, and infrastructure to ensure general tuber quality. Our results highlight the need to develop basic classification systems and quality standards for purple *D. trifida* and related crops, based on easily measurable traits like weight, size, or visual features. Such categorization could improve both experimental comparability and on-farm postharvest decision-making, especially for destructive analyses or treatment selection.

Importantly, several treatments demonstrated trait-specific advantages that could be tailored to different end uses. Integrating postharvest strategies with intended use profiles can help producers maximize the value of their harvests, particularly when working with climate-sensitive or underutilized crops.

Altogether, these findings underscore the value of simple, adaptable postharvest interventions for neglected crops like *D. trifida*. By contributing to loss reduction, nutritional preservation, and technological stability, such strategies may support rural livelihoods and food security in vulnerable areas while enhancing the visibility of underutilized biodiversity within sustainable food systems.

Further research is needed to elucidate the molecular mechanisms underlying these physiological responses, assess longer storage periods—beyond 34 days—with reduced weight losses, and evaluate potential sensory changes and effects on nutrient bioavailability associated with the tested technologies. Likewise, studies on consumer acceptance and on-farm feasibility will be key to enabling broader adoption.

This study provides a practical foundation for context-sensitive innovation in the postharvest management of underutilized roots and tubers. The approaches tested may contribute to reducing food loss, improving market access, and strengthening the role of neglected species in sustainable, agrobiodiverse food systems.

## 5. Conclusions

In *D. trifida* (purple variety tested), up to one-third of the initial weight was lost due to physical damage, microbial infection, and premature sprouting during early postharvest and transportation. Storage at 20 °C and 90% RH effectively preserved dormancy by suppressing respiration, with no sprouting observed. Among the tested strategies, cold storage at 3 °C was the most effective in minimizing weight loss, though its scalability may be limited by infrastructure costs, making it more suitable for intermediate or retail stages. Calcium dips combined with *Aloe vera* coatings helped preserve compositional quality, particularly antioxidant capacity and soluble sugars, showing a greater potential for field-level application.

Hydrothermal treatments consistently outperformed other methods in terms of practicality across supply chain stages and in preserving starch fractions, sugars, and antioxidant levels, while reducing physiological deterioration. HT at 55 °C for 5 min emerged as the most balanced protocol, combining effective quality retention with short exposure times and minimal infrastructure demands. Other protocols also provided trait-specific advantages, suggesting that HT strategies can be selected according to tuber end use and local processing goals.

## Figures and Tables

**Table 1:** Factorial 2 × 3 design for calcium dips and *Aloe vera* coatings applied to purple *D. trifida* tubers.

Treatment	I	II	III	IV	V	VI
CaCl2 (*w*/*v*)	0%	2%	1%	2%	0%	1%
Coating	None	None	None	AV-O	AV-O	AV-O

**Table 2:** Central composite rotatable design (2^2^ + star) for hydrothermal treatments at different temperatures and immersion times.

HydrothermalTreatment	HT1	HT2	HT3	HT4	HT5	HT6	HT7	HT8	HT9	HT10
Temperature (°C)	20	26	26	40.5	40.5	40.5	40.5	55	55	61
Time (min)	10	5	15	3	10	10	17	5	15	10

**Table 3:** Proximate composition (left) and mineral content (right) on a dry matter basis (mean ± SD, *n* = 3) in *D. trifida* tubers at day 1 and after 34 days of storage at 20 °C and 90% RH.

Compositional Traits (g per 100 g) ^z^	Day 1	Day 34		Day 1	Day 34
Moisture	64.74 ± 2.2 ^a^	68.23 ± 6.0 ^b^	Ca	85.04 ± 2.0 ^a^	61.50 ± 0.7 ^b^
Crude protein	5.68 ± 0.1 ^a^	5.30 ± 0.0 ^b^	Mg	74.46 ± 0.7 ^a^	63.50 ± 3.5 ^a^
Ash	2.79 ± 0.0 ^a^	2.60 ± 0.0 ^b^	K	1110.90 ± 25.3 ^a^	793.50 ± 38.9 ^a^
Crude fat	1.68 ± 0.0 ^a^	0.35 ± 0.0 ^b^	Na	30.98 ± 0.9 ^a^	12.00 ± 0.0 ^b^
Carbohydrates	81.26 ± 0.1 ^a^	87.45 ± 0.2 ^b^	Fe	20.38 ± 0.4 ^a^	6.5 ± 0.4 ^b^
Dietary fiber	8.70 ± 0.1 ^a^	9.65 ± 1.2 ^a^	Zn	1.26 ± 0.0 ^a^	1.20 ± 0.0 ^b^

^z^ Moisture was expressed on a fresh weight basis. Protein, ash, fat, and carbohydrate contents are given in g per 100 g dry sample. All minerals are given in mg per 100 g of dry sample: Ca (calcium), Mg (magnesium), K (potassium), Na (sodium), Fe (Iron), Zn (Zinc). Statistical differences between storage times (1 d and 34 d) were determined using repeated measures ANOVA with WTS (*p* ≤ 0.05). Different letters indicate significant differences, while values sharing the same letter are not significantly different.

**Table 4:** Descriptive statistics (mean ± SD, *n* = 3) for destructive and non-destructive physicochemical parameters of *D. trifida* tubers upon arrival (day 1).

Non-Destructive Physicochemical Characteristics		
Respiration rates	mL∙kg^−1^∙h^−1^ of CO_2_	6.04 ± 3.09
Weight	g	167 ± 3.50
External L*	-	42.56 ± 2.76
External a*	-	8.09 ± 0.20
External b*	-	13.39 ± 0.55
External chroma	-	15.64 ± 0.58
External hue	-	1.03 ± 0.01
**Destructive physicochemical characteristics**		
Inner L*	-	41.68 ± 1.35
Inner a*	-	22.46 ± 1.05
Inner b*	-	−3.36 ± 0.52
Inner chroma	-	22.72 ± 1.04
Inner hue	-	−0.15 ± 0.02
Firmness	Newtons	26.62 ± 5.47
Dry matter	g·kg^−1^	352.6 ± 22.2
Total soluble solids (TSS)	°Brix	6.97 ± 0.15
**Organic acids**		
Oxalic acid	mg/kg	30.12 ± 0.34
Citric acid	mg/kg	226.21 ± 4.61
Malic acid	mg/kg	91.38 ± 1.45
Succinic acid	mg/kg	871.63 ± 16.29
Acetic acid	mg/kg	1772.4 ± 614.7
**Carbohydrates**	data	data
Amylose	mg/g	26.26 ± 0.82
Amylopectin	mg/g	222.30 ± 8.33
Starch	mg/g	248.57 ± 9.13
Raffinose	mg/kg	143.96 ± 223.31
Ribose	mg/kg	0.36 ± 0.32
Sucrose	mg/kg	2216.57 ± 2467.10
Glucose	mg/kg	137.12 ± 15.48
Fructose	mg/kg	89.47 ± 31.73
**Alcohols**		
Glycerol	mg/kg	12.74 ± 11.04
**Antioxidant compounds and capacity**		
FRAP	µmol Trolox/g	13.33 ± 1.21
TEAC	µmol Trolox/g	17.62 ± 1.28
DPPH	µmol Trolox/g	80.99 ± 1.90
Folin–Ciocalteau	mg GAE/g DW	4.88 ± 0.29
Total anthocyanin content	mg Cy3Gl/100 g DW	2.38 ± 0.29

**Table 5:** Potential postharvest applications of hydrothermal treatments in *D. trifida*, based on physiological responses observed after 34 days of storage *.

Objective	Recommended HT Treatment	Effects
Antioxidant preservation	HT4 (40.5 °C—3 min)	Highest DPPH value; likely stress-induced antioxidant response
TSS minimization for frying (chips)	HT6 (40.5 °C—10 min)	Lower TSS; may prevent excessive browning during frying due to a lesser amount of reducing sugars
Starch preservation for flour	HT2 (26 °C—5 min)	Lowest respiration rate; protected amylose, amylopectin, and starch content
Enhanced sweetness for culinary uses	HT3 (26 °C—15 min)	High TSS; desirable for sweet preparations (concoctions, desserts)

* Effects include reductions in specific traits that may be of interest for targeted common uses of this kind of tuber. These associations are exploratory and derived from the current dataset.

## Data Availability

Collected data is available for public consultation on the Zenodo UPCT community through the PurpleYampeePostharvest link. The original contributions presented in the study are included in the article/Supplementary Material, further inquiries can be directed to the corresponding author.

## Supplementary Materials

The following supporting information can be downloaded at: https://www.mdpi.com/article/10.3390/foods14142436/s1, Supplementary Figure S1: preharvest defect of rejected yampees with biological defects or damage at harvest; Supplementary Figure S2: scree plot (top) and biplot (bottom) for purple yampee tubers’ characteristics during storage for 34 days at 20 °C and 90% RH); Supplementary Figure S3: contribution of each variable to the first principal cluster (PC1) among all assessed characteristics during purple yampee’s postharvest storage for 34 days at 20 °C and 90%; Supplementary Figure S4: Principal Components Analysis variables biplot (left) and contribution plot (right) for variables with interaction after repeated measures MANOVA in hydrothermally treated purple yampees; Supplementary Figure S5: sugars and alcohol evolution in cold storage temperatures. All compound concentrations are in mg/kg of purple yampee. Supplementary Table S1: statistical treatment—MANOVA analysis of characteristics after postharvest technologies applied to Purple Yampees: *p* values, resampling. Significant values are considered at *p* < 0.05.
