# Supplementary material for: Storage Behavior and Response to Low-Cost Postharvest Technologies of the Underutilized Purple Yampee (Dioscorea trifida L.f.)
Source: Foods. 2025 Jul 10;14(14):2436. doi: 10.3390/foods14142436 (PMC12294245; doi:10.3390/foods14142436)
Supplement: Supplementary file 1 [file foods-14-02436-s001.zip › foods-3715611-supplementary.pdf]

Supplementary material:

Storage behavior and response to low-cost postharvest technologies of underutilized purple yampee (*Dioscorea trifida* L.f.) by Sandra Viviana Medina-López, Jorge Andrés Jola Hernández, Maria Soledad Hernández-Gómez and Juan Pablo Fernández-Trujillo

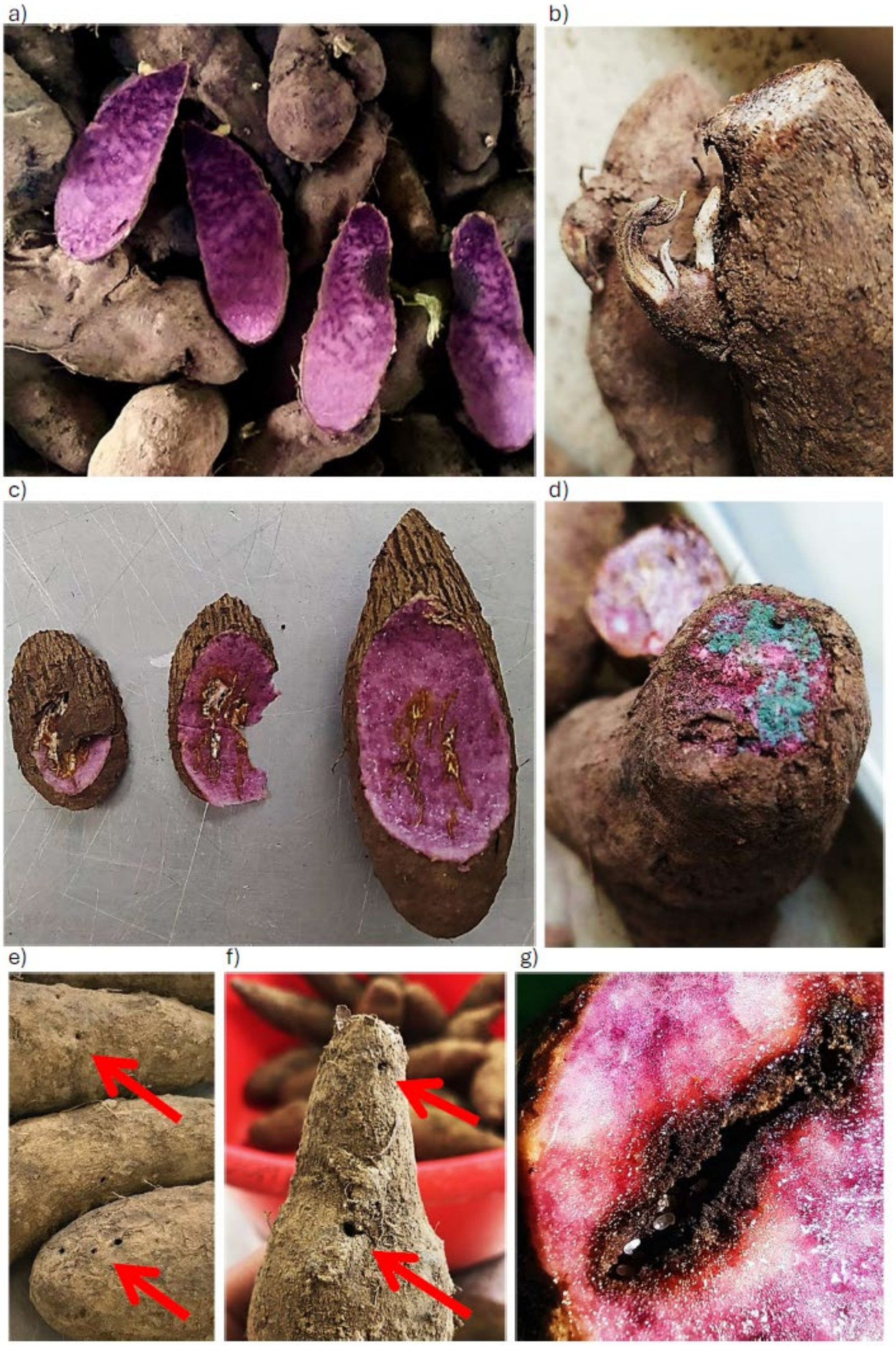

Supplementary Figure S1. Preharvest defect of rejected yampees with biological defects or damage at harvest (a-b) germinated tubers affecting morphology (c) dry rotting symptoms of necrotic tissue external and internally (d) visible fungal structures in the proximal end of a tuber (e-f) rounded holes in the surface as external signs of insect pest damage g) internal damage caused by insects.

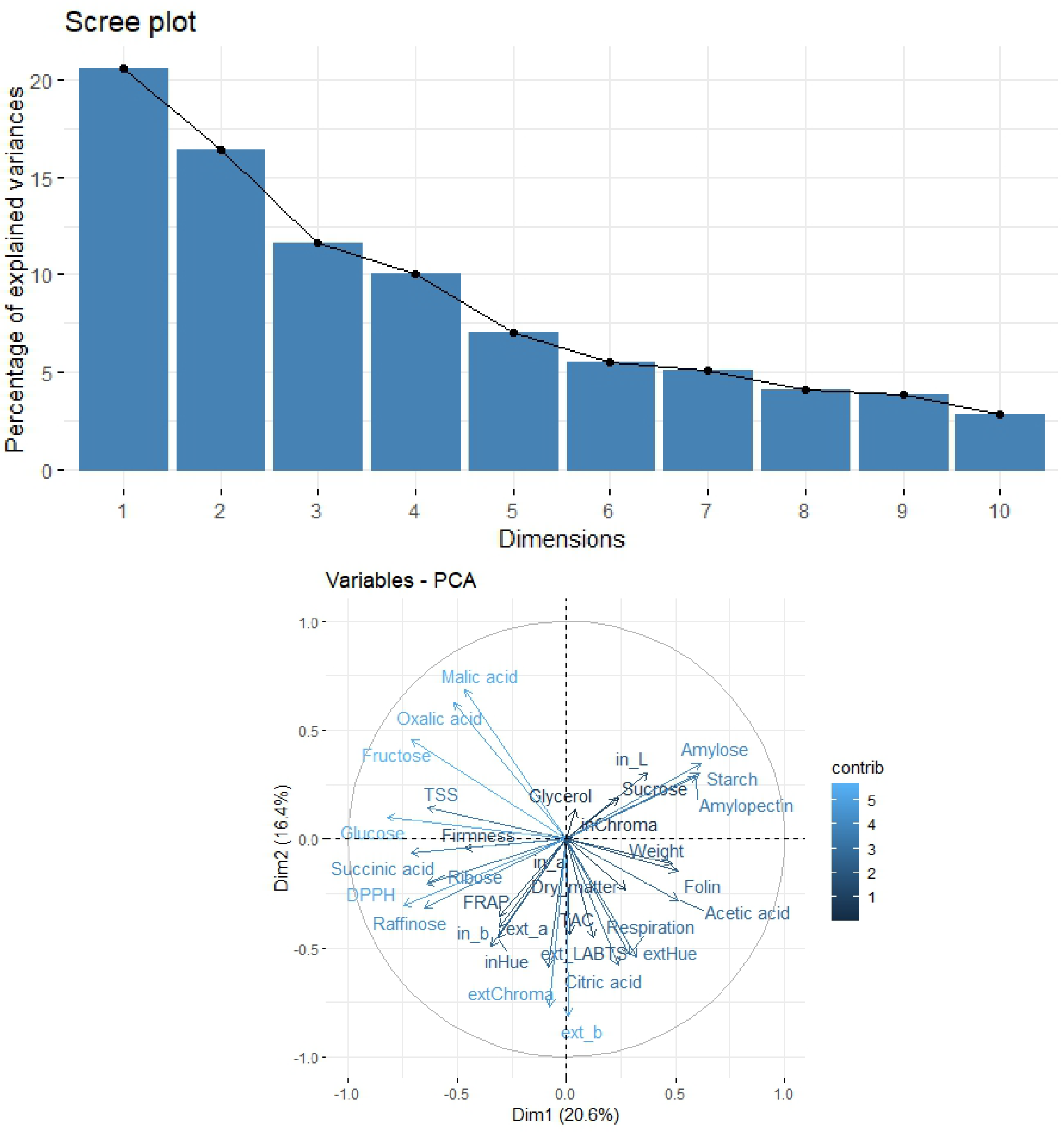

Supplementary Figure S2. Scree Plot (top) and biplot (bottom) for purple yampee tubers' characteristics during storage for 34 days at 20°C and 90% RH).

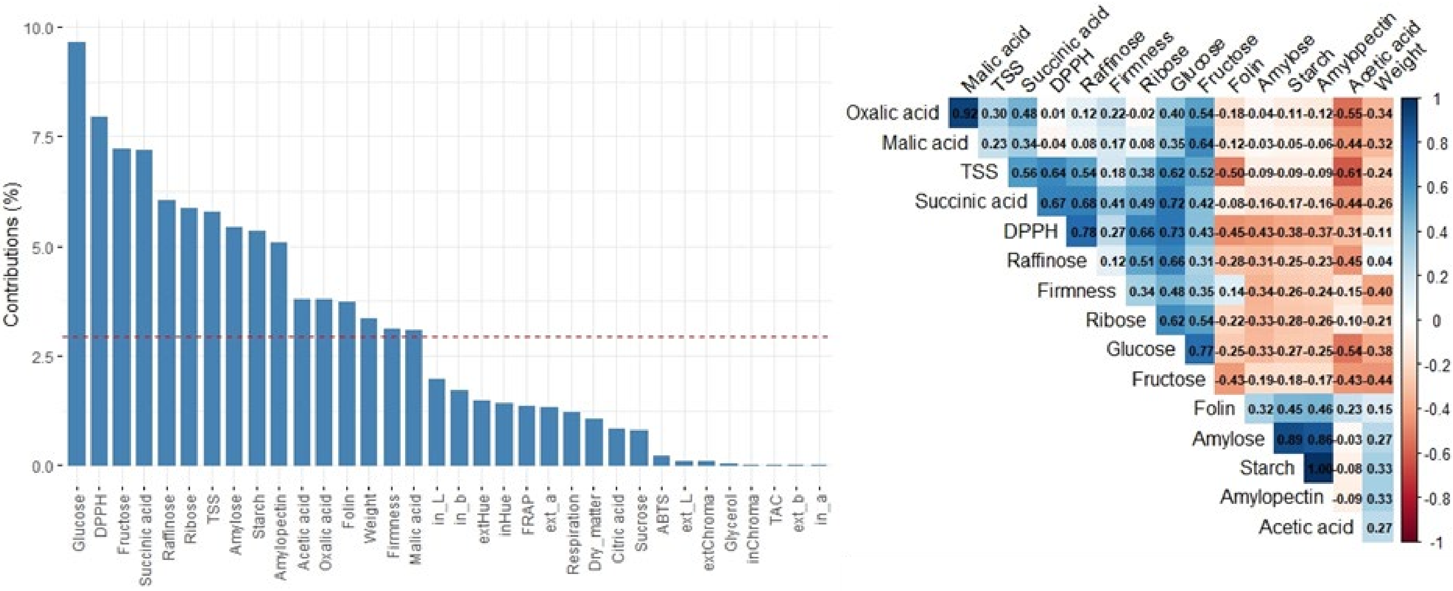

Supplementary Figure S3. Contribution of each variable to the first principal cluster (PC1) among all assessed characteristics during purple yampee's postharvest storage for 34 days at 20°C and 90% RH). (left) and correlation among variables above average contributions (right).

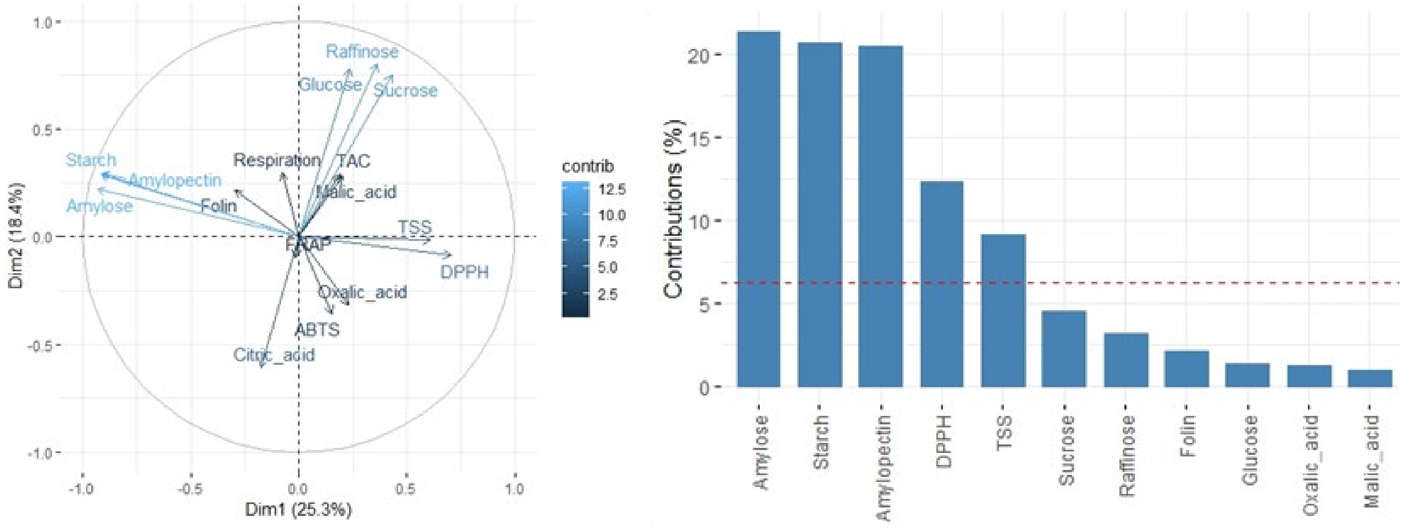

Supplementary Figure S4. Principal Components Analysis variables biplot (left) and contribution plot (right) for variables with interaction after repeated measures MANOVA in hydrothermally treated purple yampees.

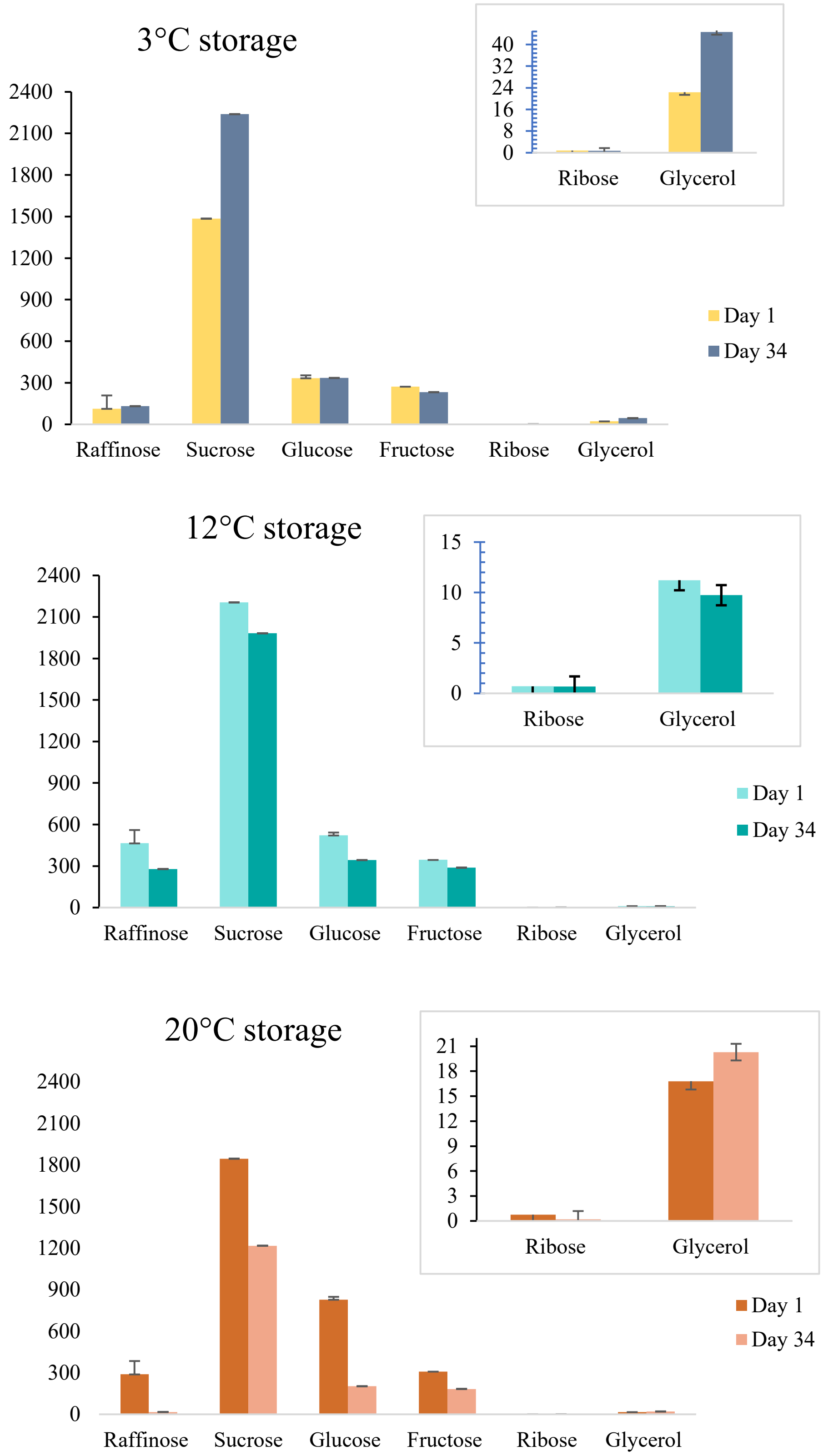

Supplementary Figure S5. Sugars and alcohol evolution in cold storage temperatures. All compounds concentrations are in mg/kg of purple yampee

Supplementary Table S1. Statistical treatment: MANOVA analysis of characteristics after postharvest technologies applied to Purple Yampees: p values, resampling. Significant values are considered at p<0.05.

Experiment	Group of variables	ParamBS (MATS) Treatment	ParamBS (MATS) Time	ParamBS (MATS) Treatment: Time
Cold storage[a]	**Antioxidants:** ABTS, FRAP, DPPH, Folin, Anthocyanins	<0.001	0.023	"<0.001
	**Sugars & alcohol**: Amylose, Amylopectin, Starch, Raffinose, Sucrose, Glucose, Fructose, Ribose, Glycerol	0.099	0.044	0.094
	**Organic acids:** Oxalic acid, Citric acid, Malic acid, Succinic acid	0.007	0.001	0.005
	**Non-destructive physicochemical:** Weight, Respiration, ext L*, ext a*, ext b*, ext Hue, ext Chroma	0.237	0.294	0.663
	**Destructive physicochemical:** in L*, in a*, in b*, in Hue, in Chroma, Firmness, Dry matter, Soluble Solids	0.128	0.047	0.189
Calcium-Coating treatments[b]	**Antioxidants:** ABTS, FRAP, DPPH, Folin, Anthocyanins	0.003	<0.001	0.011
	**Sugars & alcohol**: Amylose, Amylopectin, Starch, Raffinose, Sucrose, Glucose, Fructose, Ribose, Glycerol	0.024	<0.001	0.017
	**Organic acids:** Oxalic acid, Citric acid, Malic acid, Succinic acid	0.006	0.013	0.008
	**Non-destructive physicochemical:** Weight, Respiration, ext L*, ext a*, ext b*, ext Hue, ext Chroma	0.063	0.003	0.003
	**Destructive physicochemical:** in L*, in a*, in b*, in Hue, in Chroma, Firmness, Dry matter, Soluble Solids	0.744	0.059	0.091
Hydrothermal treatments[c]	**Antioxidants:** ABTS, FRAP, DPPH, Folin, Anthocyanins	<0.001	<0.001	<0.001
	**Sugars & alcohol**: Amylose, Amylopectin, Starch, Raffinose, Sucrose, Glucose, Fructose, Ribose, Glycerol	0.001	"<0.001	0.002
	**Organic acids:** Oxalic acid, Citric acid, Malic acid, Succinic acid	0.006	<0.001	0.004

**Non-destructive physico-chemical:** Weight, Respiration, ext L*, ext a*, ext b*, ext Hue, ext Chroma	0.871	0.021	0.816
**Destructive physicochemical:** in L*, in a*, in b*, in Hue, in Chroma, Firmness, Dry matter, Soluble Solids	0.09	<0.001	0.005

[a] Cold storage at 3, 12 and 20 ± 0.5°C (RH 84 ± 6%).

[b] Calcium-Coating treatments: sesame seed oil and *Aloe vera* gel combined with a calcium chloride treatment (0, 1 or 2% v/v).

[c] Hydrothermal treatments applied at different temperatures (20 – 61°C) and times of immersion (5 – 17 min)
